# Genome-wide screening reveals a role for subcellular localization of CRBN in the anti-myeloma activity of pomalidomide

**DOI:** 10.1038/s41598-020-61027-w

**Published:** 2020-03-04

**Authors:** Shumpei Tateno, Midori Iida, Satoshi Fujii, Tetsufumi Suwa, Miki Katayama, Haruka Tokuyama, Junichi Yamamoto, Takumi Ito, Satoshi Sakamoto, Hiroshi Handa, Yuki Yamaguchi

**Affiliations:** 1 School of Life Science and Technology, Tokyo Institute of Technology, Yokohama, 226-8501 Japan; 2 School of Computer Science and Systems Engineering, Kyushu Institute of Technology, Iizuka, 820-0067 Japan; 3 Department of Chemical Biology, Tokyo Medical University, Shinjuku, 160-8402 Japan

**Keywords:** Chemotherapy, Ubiquitylation, Target identification, Molecular medicine

## Abstract

Pomalidomide, a derivative of thalidomide, is an effective treatment for multiple myeloma. The drug exerts its effects through CRBN, a component of the E3 ubiquitin ligase complex CRL4^CRBN^. To search for novel factors involved in the anti-cancer activity of pomalidomide, we performed a genome-wide shRNA library screen and identified 445 genes as those affecting pomalidomide sensitivity. Genes encoding components of the ubiquitin-proteasome pathway, such as subunits of the CRL4^CRBN^ complex, the COP9 signalosome, and the 26S proteasome, were among the pomalidomide-affecting genes. Karyopherin beta 1 (*KPNB1*) was identified as a novel pomalidomide-affecting gene. KPNB1 was required for the nuclear import of CRBN and for the CRBN-directed, pomalidomide-dependent degradation of a clinically relevant substrate, the transcription factor Aiolos. By contrast, the cytoplasmic translation factor GSPT1 was degraded following treatment with the thalidomide derivative CC-885 only when CRBN was present in the cytoplasm, indicating that subcellular distribution of CRBN is critical for the efficacy of thalidomide-based medications.

## Introduction

Thalidomide was developed as a sedative in the 1950s but was withdrawn from the market due to its teratogenicity. However, subsequent studies revealed that thalidomide possesses immunomodulatory effects and has therapeutic potential against leprosy and cancer^1,2^. Thalidomide was approved for the treatment of erythema nodosum leprosum in 1998 and for the treatment of multiple myeloma in 2006. In parallel, thalidomide derivatives such as lenalidomide, pomalidomide, and CC-885 have been developed, and in the mid-2000s, lenalidomide was approved for treatment of multiple myeloma and 5q deletion-associated myelodysplastic syndrome (5q^–^ syndrome). In 2013, pomalidomide was also approved as a cure for multiple myeloma. CC-885 is a newly developed compound with potent antitumor activity against not only hematological cancers but also epithelial cancers^3^. Thus, several thalidomide derivatives have been used to treat various diseases, and their pharmacological value is anticipated to expand further in the future.

CRBN is a primary target of thalidomide and is responsible for the various pharmacological activities of the drug and related compounds^1,4,5^. Collectively, thalidomide and its derivatives are considered immunomodulatory drugs or CRBN modulators. CRBN is a component of the E3 ubiquitin ligase complex that also contains DDB1, CUL4, and ROC1. As the substrate receptor, CRBN recruits substrates to the CRL4^CRBN^ complex and induces polyubiquitination and degradation by the 26S proteasome. Cullin-based ubiquitin ligase activity is stimulated by neddylation (*i.e*., NEDD8 conjugation) to cullin and downregulated by deneddylation mediated by the COP9 signalosome^6,7^. CRBN modulators bind to CRBN and elicit their effects by altering its substrate specificity. For example, lenalidomide and pomalidomide induce CRBN-dependent ubiquitination and degradation of Ikaros and Aiolos, transcription factors required for the development of lymphoids and survival of multiple myeloma^8–10^. Lenalidomide also induces degradation of CK1α, a clinical target of 5q^–^ syndrome^11,12^. On the other hand, CC-885 induces degradation of the translation factor GSPT1, thereby eliciting broad-spectrum growth inhibition against a variety of cancer cell lines^3^. Moreover, thalidomide-induced degradation of the transcription factors p63 and SALL4 has been implicated in embryopathy^13–15^. Thus, each CRBN modulator acts as a molecular “glue” that mediates the interaction between CRBN and specific substrates^16^. In other words, CRBN enables its various modulators to induce their pharmacological effects by recruiting specific substrates to CRL4^CRBN^.

Identification of factors that regulate or are regulated by CRBN is extremely important because knowledge of these proteins could help elucidate the mechanism of action of CRBN modulators and facilitate the development of new thalidomide derivatives. Attempts to identify such factors have been made previously, almost entirely using biochemical approaches, leading to the discovery of a number of CRBN substrates. Given the complexity of pharmacological activities of CRBN modulators, however, many unidentified CRBN substrates are likely to exist. Moreover, the regulation of CRL4^CRBN^ remains largely unknown and may involve novel interactors or post-translational modifications. To address these questions, an unbiased comprehensive approach is needed. In this study, we performed a genome-wide shRNA screen to identify genes affecting the anti-myeloma activity of pomalidomide.

## Results

### Identification of pomalidomide-affecting genes by shRNA library screening

**Figure 1:**
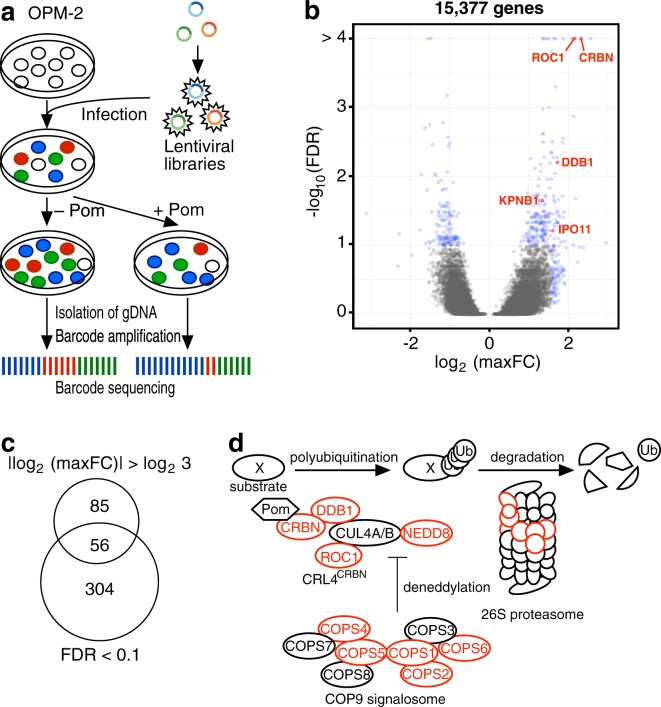
shRNA library screen for discovery of pomalidomide-affecting genes. (**a**) Outline of shRNA library screen. OPM-2 cells were infected with lentiviral shRNA libraries, split, and cultured with or without 1 μM pomalidomide for 5 days. Genomic DNA was extracted, and the abundance of each barcode, representing the number of cells expressing each shRNA, was quantified by high-throughput sequencing. (**b**) Scatter plot showing the results of shRNA library screening. Blue dots represent pomalidomide-affecting genes. Red dots represent CRL4^CRBN^ subunits and importin-β family members identified by the screen. (**c**) Venn diagram showing the number of pomalidomide-affecting genes identified by two statistical criteria. (**d**) Illustration depicting the CRBN-dependent ubiquitin-proteasome pathway and its regulatory factors. Genes identified by the screen are colored in red.

To identify genes involved in the anti-cancer effect of pomalidomide on multiple myeloma, we performed genome-wide screening using pooled shRNA libraries containing 82,500 shRNAs targeting 15,377 human genes. Specifically, we infected OPM-2 multiple myeloma cells with lentiviral shRNA libraries, treated the infected cells with pomalidomide or vehicle, and then performed high-throughput sequencing of 18 bp barcode sequences to estimate the number of surviving cells that had received each shRNA (Fig. 1a and Supplementary Table 1). We then deduced the pomalidomide sensitivity of the knockdown cells from the relative number of reads per million reads of the corresponding barcodes from pomalidomide-treated vs. untreated samples. Two different statistical values, false discovery rate (FDR) and maxFC, were calculated for each gene and used to identify genes affecting pomalidomide sensitivity (Fig. 1b). Based on FDR and maxFC, 445 genes were identified by at least one criterion: 141 genes had |log_2_(maxFC)| > log_2_(3) in pomalidomide-treated vs. untreated samples, and 360 genes had FDR < 0.1; 56 genes met both criteria (Fig. 1c and Supplementary Table 2). As expected, subunits of the CRBN complex, including CRBN, ROC1, and DDB1, were included among the set of overlapping genes (Fig. 1d and Supplementary Table 2). In addition, other genes involved in the ubiquitin-proteasome pathway, such as NEDD8, components of the COP9 signalosome, and components of the 26 S proteasome, were enriched among the 445 genes meeting at least one criterion (Fig. 1d and Supplementary Table 2). Thus, our screening results confirmed and extended previous biochemical findings that the CRL4^CRBN^ complex is a primary pharmacological target of CRBN modulators.

**Figure 2:**
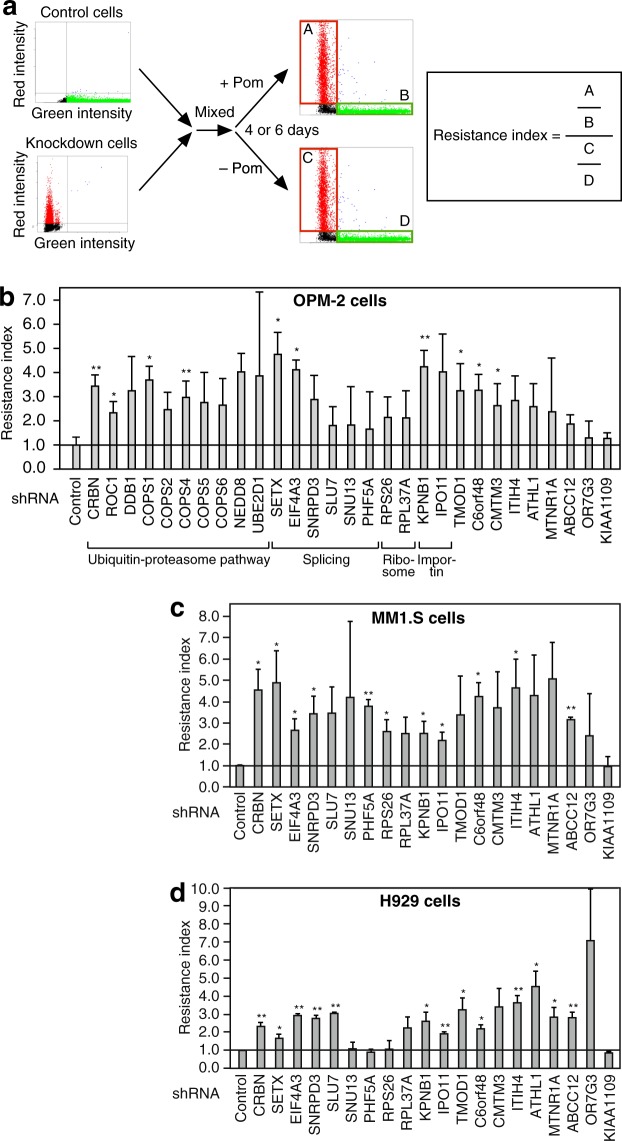
Validation experiments for Pomalidomide-affecting genes. (**a**) Calculation of the resistance index. An equal number of shRNA-transduced RFP-positive cells and control GFP-positive cells were mixed and incubated for 4 (OPM-2) or 6 (MM1.S and H929) days in the presence or absence of 1 µM pomalidomide (Pom). Fluorescent live cells were counted using an image-based cytometer, and the resistance index was calculated as indicated. (**b–d**) Bar graphs indicating the resistance indices of shRNAs identified by the screen. Error bars represent standard deviations (S.D.) (n = 3). *P < 0.05, **P < 0.01 (Student’s t-test).

To validate these results, we selected shRNAs targeting 29 genes, including *CRBN*, all of which conferred resistance to pomalidomide in the screen. Knockdown efficiency of the individual shRNAs employed was evaluated by quantitative RT-PCR following transduction of lentiviral vectors expressing shRNAs into OPM-2 cells (Supplementary Fig. 1). For the validation experiment, an equal number of shRNA-transduced RFP-positive cells and control GFP-positive cells were mixed and incubated for 6 days in the presence of pomalidomide. Fluorescent live cells were counted on an image-based cytometer, and the resistance index was calculated as described in Fig. 2a. If knockdown conferred resistance to pomalidomide, the resistance index would be greater than 1. Most of the shRNAs tested conferred resistance to pomalidomide (Fig. 2b). To further validate the above results, we repeated the experiments using a subset of the shRNAs in two other multiple myeloma cell lines, namely MM1.S and H929. Since components of CRL4^CRBN^ and the COP9 signalosome were independently identified in similar genetic screens using other cell lines^17,18^, they were removed from further analysis. Consequently, similar results were obtained (Fig. 2c,d), supporting our conclusion that many of the genes identified by our screen are indeed involved in pomalidomide activity.

### KPNB1 is important for nuclear import of CRBN and pomalidomide activity

**Figure 3:**
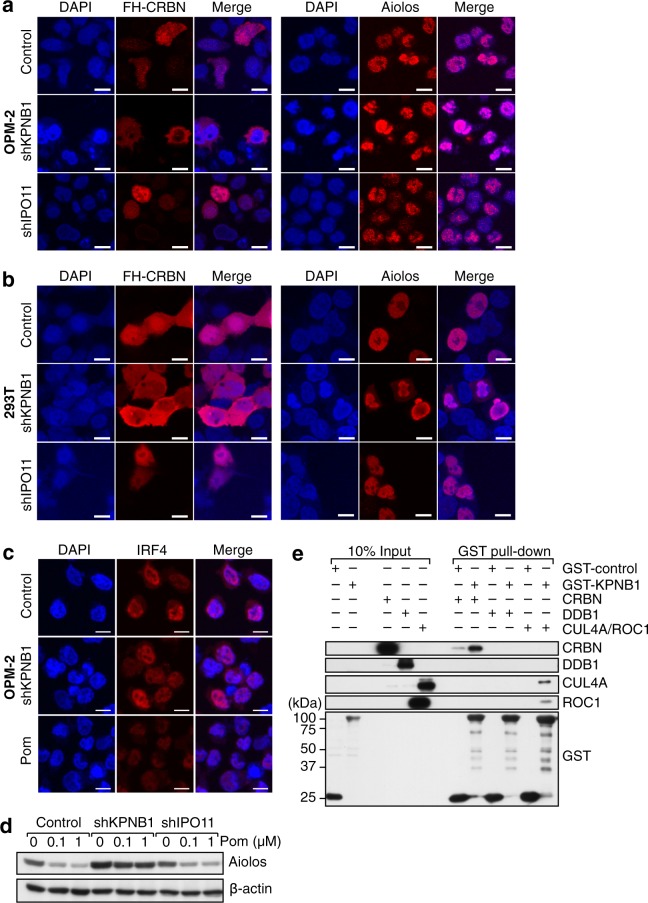
KPNB1, but not IPO11, is important for nuclear import of CRBN. (**a**,**c**) Immunofluorescence microscopy analysis of CRBN-knockout OPM-2 cells stably expressing FLAG-HA (FH)-CRBN. Following lentiviral transduction of shRNA targeting KPNB1 or IPO11, or treatment with 1 µM pomalidomide for 48 h, the cells were stained with anti-HA, anti-Aiolos, or anti-IRF4 antibody (red) and counterstained with DAPI (blue). Scale bar, 10 µm. (**b**) Immunofluorescence microscopy analysis was performed using CRBN-knockout 293 T cells stably expressing FH-CRBN. Following transient transfection of Aiolos- and shRNA-expression vectors, the resultant cells were subjected to immunofluorescence staining. (**d**) Immunoblot analysis of 293 T cells transiently expressing Aiolos and the indicated shRNAs. The cells were treated with the indicated concentrations of pomalidomide for 12 h before analysis. **e**, Control GST or GST-KNPB1 coupled to glutathione beads was incubated with lysates of High Five insect cells overproducing the indicated proteins. Input (10%) and bound proteins were subjected to immunoblot analysis.

To search for novel factors involved in pomalidomide activity, we performed Gene Ontology (GO) enrichment analysis using Database for Annotation, Visualization and Integrated Discovery DAVID (Table 1). Unexpectedly, biological processes related to gene expression, such as translation (GO:0006412) and RNA splicing (GO:008380), were highly enriched. In addition, members of the importin-β family^19^, such as importin 11 (IPO11) and karyopherin beta 1 (KPNB1), were also enriched (GO:0006610). Hence, we investigated whether knockdown of KPNB1 or IPO11 would affect the subcellular localization of CRBN or Aiolos, a pharmacologically relevant substrate of CRBN. We made CRBN-knockout OPM-2 cells using the CRISPR/Cas9 system and transduced them with a lentiviral vector expressing FLAG- and HA-tagged CRBN (FH-CRBN) for imaging. Immunofluorescence microscopy revealed that under control conditions, FH-CRBN was present in both the nucleus and the cytoplasm, whereas Aiolos was localized in the nucleus, *i.e*., CRBN was partially colocalized with Aiolos in the nucleus (Fig. 3a). We then knocked down KPNB1 and IPO11 individually. Strikingly, the nuclear fraction of FH-CRBN was decreased by knockdown of KPNB1 but not by knockdown of IPO11 (Fig. 3a). On the other hand, nuclear localization of Aiolos was not affected by either knockdown. To confirm that these results were not specific to OPM-2 cells, we repeated the experiments using 293 T cells transiently overexpressing Aiolos and obtained essentially the same results (Fig. 3b). Moreover, KPNB1 knockdown had little effect on the nuclear localization of IRF4, a downstream mediator of pomalidomide-induced growth inhibition, in OPM-2 cells (Fig. 3c). Thus, KPNB1 is critical for the nuclear import of CRBN, but not of Aiolos and IRF4, and CRBN does not spatially overlap with its substrate Aiolos in the absence of KPNB1.

**Table 1:** Enriched Biological Processes from GO analysis of pomalidomide-affecting genes.

GO accession	GO term: Biological Process	-log10[P-value]	Genes
GO:0006413	translational initiation	16.2	COPS5, RPL14, RPL35, RPL36, RPL23A, RPS26, EIF3D, EIF4G2, EIF4G3, RPL30, EIF3B, RPL7, RPL32, RPL23, RPL31, RPL6, RPS14, EIF2S2, RPL8, RPS13, RPL10, RPL5, RPL37A, RPS11
GO:0006614	SRP-dependent cotranslational protein targeting to membrane	13.8	RPL14, RPL35, RPL36, RPL23A, SRP19, RPS26, RPL30, RPL7, RPL32, RPL23, RPL31, RPL6, RPS14, RPL8, RPL10, RPS13, RPL5, RPL37A, RPS11
GO:0006364	rRNA processing	12.8	RPL14, SNU13, RPL35, RPL36, SKIV2L2, RPS26, RPL30, DDX47, RPL7, RPL32, RPL6, RPL31, RPL8, RPL10, RPL5, RPL23A, PWP2, EIF4A3, RPL23, RPS14, RPS13, WDR3, RPL37A, RPS11, C1D
GO:0000184	nuclear-transcribed mRNA catabolic process, nonsense-mediated decay	12.0	RPL14, RPL35, RPL36, RPL23A, RPS26, EIF4A3, RPL30, RPL7, RPL32, RPL23, RPL31, RPL6, RPS14, RPL8, RPS13, RPL10, RPL5, RPL37A, RPS11
GO:0000398	mRNA splicing, via spliceosome	11.6	PABPN1, CRNKL1, U2AF2, SNRPD3, SNU13, SNRPD1, SNRPD2, SKIV2L2, SF3A1, SNURF, POLR2A, EIF4A3, AQR, HNRNPK, USP39, SNRNP200, DHX15, SLU7, USP49, PHF5A, HNRNPC, DDX41, SNRPE, SNRPG
GO:0019083	viral transcription	11.3	RPL14, RPL35, RPL36, RPL23A, RPS26, RPL30, RPL7, RPL32, RPL23, RPL31, RPL6, RPS14, RPL8, RPL10, RPS13, RPL5, RPL37A, RPS11
GO:0006412	translation	9.6	COPS5, RPL14, RPL35, SNU13, RPL36, RPL23A, IGHMBP2, SLC25A30, RPS26, RPL30, RPL7, RPL32, RPL23, RPL31, RPL6, RPS14, RPL8, RPS13, RPL10, RPL5, RPL37A, RPS11, TNIP1
GO:0000715	nucleotide-excision repair, DNA damage recognition	5.8	GPS1, COPS2, COPS5, COPS6, DDB1, COPS4, RBX1
GO:0000245	spliceosomal complex assembly	5.5	CRNKL1, SNRPD3, USP39, SNRPD1, SNRPD2, SNRPE, SNRPG
GO:0006283	transcription-coupled nucleotide-excision repair	5.4	GPS1, COPS2, UVSSA, AQR, COPS5, COPS6, DDB1, COPS4, POLR2A, RBX1
GO:0010388	cullin deneddylation	5.1	GPS1, COPS2, COPS5, COPS6, COPS4
GO:0038061	NIK/NF-kappaB signaling	4.9	PSMA2, PSMA1, PSMC6, PSMA6, PSMA5, PSMA3, PSMD1, RIPK3, SKP1
GO:0090263	positive regulation of canonical Wnt signaling pathway	4.5	PSMA2, PSMA1, PSMC6, PSMA6, PSMA5, UBR5, PSMA3, ZBED3, PSMD1, LGR4, WNT2B
GO:0002223	stimulatory C-type lectin receptor signaling pathway	4.2	PSMA2, UBE2N, PSMA1, PSMC6, PSMA6, PSMA5, PSMA3, PSMD1, SKP1, TAB1
GO:0006369	termination of RNA polymerase II transcription	4.0	PABPN1, EIF4A3, SNRPD3, U2AF2, SLU7, SNRPE, SETX, SNRPG
GO:0008380	RNA splicing	4.0	EIF4A3, DDX47, SON, SNRPD3, USP39, SNRPD1, DHX15, SNRPD2, HNRNPC, WTAP, SNURF, SNRPG
GO:0043161	proteasome-mediated ubiquitin-dependent protein catabolic process	3.8	PSMA2, PSMA1, PSMC6, CRBN, KBTBD4, PSMA6, PSMA5, ARRB1, DDB1, PSMA3, PSMD1, SKP1, RBX1
GO:0050852	T cell receptor signaling pathway	3.7	PSMA2, UBE2N, PSMA1, PTPRC, PSMC6, PSMA6, PSMA5, PSMA3, PSMD1, SKP1, RFTN1
GO:0051170	nuclear import	3.7	SNRPD3, SNRPD1, SNRPD2, SNRPE, SNRPG
GO:0006521	regulation of cellular amino acid metabolic process	3.7	PSMA2, PSMA1, PSMC6, PSMA6, PSMA5, PSMA3, PSMD1
GO:0006396	RNA processing	3.7	PABPN1, HNRNPK, CRNKL1, SNRPD3, ADAD1, SNRPD1, DDX54, SF3A1, SETX
GO:0000209	protein polyubiquitination	3.6	PSMA2, PSMA1, LRSAM1, PSMC6, PSMA6, PSMA5, UBR5, PSMA3, PSMD1, SKP1, RNF14, RBX1
GO:0051437	positive regulation of ubiquitin-protein ligase activity involved in regulation of mitotic cell cycle transition	3.6	PSMA2, PSMA1, PSMC6, PSMA6, PSMA5, PSMA3, PSMD1, SKP1
GO:0016055	Wnt signaling pathway	3.5	RSPO4, WNT7B, AMER2, LDB1, DDB1, RTF1, ZBED3, PAF1, SKP1, CSNK1G3, RBX1, WNT2B
GO:0002479	antigen processing and presentation of exogenous peptide antigen via MHC class I, TAP-dependent	3.2	PSMA2, PSMA1, PSMC6, PSMA6, PSMA5, PSMA3, PSMD1
GO:0002181	cytoplasmic translation	3.1	RPL7, RPL6, RPL31, RPL8, RPL36
GO:0038095	Fc-epsilon receptor signaling pathway	3.1	PSMA2, UBE2N, PSMA1, PSMC6, PSMA6, PSMA5, PSMA3, PSMD1, MS4A2, SKP1, TAB1
GO:0006511	ubiquitin-dependent protein catabolic process	3.0	PSMA2, UBE2N, PSMA1, PSMC6, PSMA6, USP9X, UBR5, PSMA3, NEDD8, USP49, SKP1
GO:0000387	spliceosomal snRNP assembly	3.0	SNRPD3, SNRPD1, SNRPD2, SNRPE, SNRPG
GO:0051436	negative regulation of ubiquitin-protein ligase activity involved in mitotic cell cycle	2.9	PSMA2, PSMA1, PSMC6, PSMA6, PSMA5, PSMA3, PSMD1
GO:0000338	protein deneddylation	2.8	COPS5, COPS6, COPS4
GO:0090090	negative regulation of canonical Wnt signaling pathway	2.8	PSMA2, PSMA1, PSMC6, AMER2, PSMA6, PSMA5, PSMA3, PSMD1, WWTR1, RBX1
GO:0031145	anaphase-promoting complex-dependent catabolic process	2.7	PSMA2, PSMA1, PSMC6, PSMA6, PSMA5, PSMA3, PSMD1
GO:0051301	cell division	2.6	KIF11, MAU2, USP9X, BIRC6, KNSTRN, REEP4, CCNB3, RAD21, PPP1R1C, USP39, KIF20B, SPG20, ARL8B, NSUN2, TUBA1B
GO:0060071	Wnt signaling pathway, planar cell polarity pathway	2.4	PSMA2, PSMA1, PSMC6, PSMA6, PSMA5, PSMA3, PSMD1
GO:0000027	ribosomal large subunit assembly	2.3	RPL6, RPL10, RPL5, RPL23A
GO:0006610	ribosomal protein import into nucleus	2.1	RPL23, IPO11, KPNB1
GO:0043488	regulation of mRNA stability	2.1	PSMA2, PSMA1, PSMC6, PSMA6, PSMA5, PSMA3, PSMD1

To determine whether KPNB1 affects pomalidomide-induced degradation of Aiolos, we treated KPNB1 knockdown cells with pomalidomide and determined the Aiolos protein level by immunoblotting (Fig. 3d). Although Aiolos was substantially degraded following pomalidomide treatment in control cells, the Aiolos level was not significantly decreased by pomalidomide in KPNB1 knockdown cells. Collectively, these data indicate that KPNB1 contributes to the pomalidomide-induced degradation of Aiolos by facilitating nuclear import of CRBN.

To gain insight into the mechanism by which KPNB1 controls subcellular localization of CRBN and regulates pomalidomide activity, we examined protein-protein interactions of KPNB1 with subunits of CRL4^CRBN^. Pull-down assays using GST-tagged KPNB1 and CRL4^CRBN^ subunits individually overproduced using the baculovirus expression system showed that KPNB1 interacts with CRBN and, to a lesser extent, CUL4A and ROC1 (Fig. 3e), suggesting that KPNB1 mediates nuclear import of CRBN through direct, and possibly indirect interactions.

### Subcellular localization of CRBN affects the efficacy of CRBN modulators

**Figure 4:**
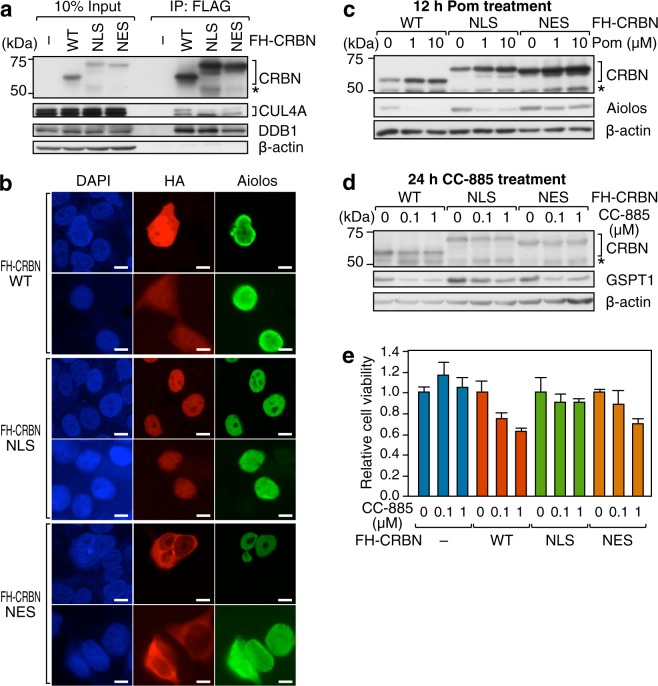
Spatial overlaps of CRBN and its substrates are important for the efficacy of CRBN modulators. (**a**) CRL4^CRBN^ was immunoprecipitated (IP) using anti-FLAG beads from CRBN-knockout 293 T cells expressing FH-CRBN, FH-NLS-CRBN, or FH-NES-CRBN, and their subunit compositions were examined by immunoblot analysis. Asterisks denote nonspecific signals. (**b**) Immunofluorescence microscopy analysis of CRBN-knockout 293 T cells transiently expressing Aiolos and FH-CRBN. Cells were stained with anti-HA (red), anti-Aiolos (green), and DAPI (blue). Scale bar, 10 µm. (**c,d**) CRBN-knockout 293 T cells expressing Aiolos and FH-CRBN, FH-NLS-CRBN, or FH-NES-CRBN were treated with the indicated concentrations of pomalidomide for 12 h (**c**) or CC-885 for 24 h (**d**), and then subjected to immunoblot analysis using the indicated antibodies. (**e**) CRBN-knockout 293 T cells and the knockout cells re-expressing wild-type (WT), NLS-fused, or NES-fused CRBN were treated with the indicated concentrations of CC-885 for 48 h. Cell viability was assayed, normalized against the number of untreated cells, and displayed as the mean ± S.D. of three independent experiments.

The results described above suggest that the colocalization of CRBN and its substrate is critical for the efficacy of pomalidomide. To extend this idea, we fused CRBN to the nuclear localization signal (NLS) derived from the SV40 large T antigen or to the nuclear export signal (NES) derived from HIV-1 Rev^20,21^. We then transduced CRBN-knockout 293T cells with wild-type, NLS-fused, or NES-fused CRBN. Coimmunoprecipitation study showed that ectopically expressed CRBN proteins were incorporated into CUL4-based E3 ligase complexes (Fig. 4a). Immunofluorescence microscopy analysis revealed that a fraction of both wild-type and NLS-fused CRBN was imported into the nucleus and overlapped with Aiolos (Fig. 4b). By contrast, NES-fused CRBN was found only in the cytoplasm and did not overlap with Aiolos. When these cells were treated with pomalidomide, Aiolos was degraded in cells expressing wild-type or NLS-fused CRBN, but not in cells expressing NES-fused CRBN (Fig. 4c), indicating that CRBN in the nucleus is required for Aiolos degradation. When these cells were treated with CC-885, GSPT1, a CC-885–dependent CRBN substrate localized in the cytoplasm, was degraded in cells expressing wild-type or NES-fused CRBN, but not in cells expressing NLS-fused CRBN (Fig. 4d), indicating that CRBN in the cytoplasm is required for its degradation. Concordantly, cells expressing wild-type or NES-fused CRBN were susceptible to growth inhibition by CC-885, whereas cells expressing NLS-fused CRBN were as resistant to CC-885 as parental CRBN-knockout cells (Fig. 4e). These contrasting results strongly suggest that the spatial overlap of CRBN and its substrates is important for their efficient ubiquitination and degradation.

## Discussion

Our screen identified 445 pomalidomide-affecting genes, including CRBN and other components of the ubiquitin-proteasome pathway, such as ROC1, DDB1, NEDD8, subunits of the COP9 signalosome, and subunits of the 26S proteasome. In most cases, individual knockdown experiments replicated the results obtained from the screen, suggesting that our screen was successful. To our surprise, GO analysis revealed that factors involved in nuclear export and the gene expression pathways, such as RNA splicing and translation, are also involved in anti-myeloma activity of pomalidomide. Moreover, we found that subcellular localization of CRBN is controlled by KPNB1 and is important for sensitivity to CRBN modulators. Our findings suggest new approaches for strengthening or controlling the efficacy of CRBN modulators by regulating the subcellular localization of CRBN.

Two recent studies independently conducted genome-wide CRISPR knockout screens to identify factors affecting the efficacy of lenalidomide^17,18^. Concordant with our findings, a number of COP9 signalosome subunits were identified by Sievers *et al*.^17^, supporting the idea that an appropriate level of CUL4 neddylation is necessary in order for CRL4^CRBN^ to mediate the efficacy of lenalidomide and pomalidomide (Fig. 1d). The two studies also identified UBE2D3 and UBE2G1 as E2 ubiquitin-conjugating enzymes specific to CRL4^CRBN^. By contrast, our shRNA screen did not identify UBE2D3 and UBE2G1. One reason for this discrepancy might be that shRNAs targeting these genes were unable to cause discernible phenotypes due to the genes’ partially redundant functions. On the other hand, KPNB1 was identified only in our screen.

Importin-β family members recognize a specific region of cargo proteins called the NLS. The importin-β family includes at least 22 members in humans, and each member is thought to recognize and import a partially overlapping but distinct set of cargo proteins^19^. We showed that KPNB1 is critical for the nuclear import of CRBN, but not of Aiolos and IRF4 (Fig. 3a–c), suggesting that CRL4^CRBN^ is a primary cargo of KPNB1 in the context of regulating pomalidomide activity. We also showed that KPNB1 directly interacts with CRBN (Fig. 3e). Using PSORT II, a program for predicting protein subcellular localization sites^22^, no putative NLS was identified in CRBN; however, CRBN was predicated to be nuclear using both PSORT II and PredictNLS. For CRL4^DDB2^, the CRL4-based E3 ligase complex into which DDB2 is incorporated as a substrate receptor, DDB2 plays a key role in its nuclear translocation and accumulation to the sites of DNA damage in response to UV irradiation^23^. Similarly, CRBN may play a determinant role in the subcellular localization of CRL4^CRBN^, although we cannot exclude the possibility that KPNB1 imports CRBN indirectly through an interaction with another component of CRL4^CRBN^.

In addition, we showed that spatial overlap between CRBN and its substrates is important for efficient degradation of the substrates and for the efficacy of CRBN modulators. To date, over a dozen proteins including Ikaros, Aiolos, GSPT1, CK1α, glutamine synthetase, MEIS2, KCNMA1/SLO1, CLC1, and AMPKα have been identified as substrates for CRBN^3,11–16,24–28^. The subcellular localizations of these factors differ: the transcription factors Ikaros, Aiolos, and MEIS2 are localized to the nucleus, whereas GSPT1, CK1α, and glutamine synthetase reside in the cytoplasm. The potassium channel KCNMA1/SLO1 and the chloride channel CLC1 are transmembrane proteins whose CRBN-mediated degradation is thought to occur during membrane trafficking^26,27^. Meanwhile, the subcellular distribution of CRBN also varies among cell lines. In OPM-2 and 293T cells, we detected CRBN in both the cytoplasm and the nucleus, with slight accumulation in the nucleus (Fig. 3a,b). In DF15 cells, however, CRBN appears to reside largely in the cytoplasm^5^. It is therefore plausible that subcellular distribution of CRBN is influenced by the pathological or physiological conditions of the cell. It is even possible that subcellular distribution of CRBN affects the efficiency of ubiquitination and degradation of its substrates in response to various CRBN modulators. For example, cytoplasmic anchorage of CRBN, and the resulting sequestration from Ikaros and Aiolos may attenuate their degradation in response to lenalidomide or pomalidomide, thereby providing a mechanism by which multiple myeloma cells can acquire resistance to these drugs. Thus, finding ways to control the subcellular distribution of CRBN could help to improve the efficacy of CRBN modulators.

How individual genes identified by our screen affect pomalidomide sensitivity remains largely unknown and is the subject of our future study. Splicing factors, such as SNRPD3, SLU7, and PHF5A, and ribosomal proteins, such as RPS26 and RPL37A, may be necessary for production of factors involved in pomalidomide-induced growth inhibition, such as CRBN, Ikaros, Aiolos, IRF4, and MYC. On the other hand, *ABCC12*, one of the genes identified by our screen, is a poorly characterized member of the ATP-binding cassette transporter superfamily^29^. Since ABCC subfamily members are thought to confer multidrug resistance to tumor cells, ABCC12 may affect pomalidomide sensitivity by controlling the efflux of pomalidomide. The precise role of IPO11 is also unclear. Since IPO11 is dispensable for the nuclear import of CRBN and Aiolos (Fig. 3a,b), IPO11 is likely involved in the transport of another nuclear factor critical for pomalidomide-induced growth inhibition.

## Methods

### Cell culture

OPM-2, MM1.S, and H929 cells were maintained in RPMI 1640 with 10% FBS, 1 mM sodium pyruvate, 2.5 mM glucose, and antibiotics. 293T cells were maintained in DMEM with 10% FBS and antibiotics. CRBN-knockout cells were obtained from OPM-2 and 293T cells by transfecting a pX330-based CRISPR/Cas9 vector^30^ targeting the first exon of *CRBN*, and treating the cells with 0.1 µM CC-885 for 2 weeks to eliminate cells harboring wild-type *CRBN*. OPM-2 cells were transfected using a Nucleofector 2b device and the A-020 program (Lonza). 293T cells were transfected using Lipofectamine 2000 (Thermo Fisher Scientific). FH-CRBN, with or without NLS and NES, was transduced into OPM-2 cells using the lentiviral system or into 293T cells using Lipofectamine 2000.

### shRNA library screening

The scheme for the high-throughput shRNA library screen is shown in Fig. 1a. Human Modules 1, 2, and 3 of pooled bar-coded lentiviral shRNA libraries (Cellecta), each containing 27,500 shRNAs targeting about 5,000 genes (~5 shRNAs per gene), were used individually for screening. Plasmid libraries were packaged using the ViraPower Lentiviral Packaging Mix (Thermo Fisher Scientific). OPM-2 cells (3.0 × 10^7^) were infected with the resultant lentiviral libraries at a multiplicity of infection of 0.3. Three days after infection, the cells were divided and treated with 1 µM pomalidomide or left untreated for 5 days. Then, extraction of genomic DNA and preparation of sequencing libraries were performed as described in the Cellecta’s user manual. Barcodes representing individual shRNAs were quantified by high-throughput sequencing on a HiSeq. 2000 (Illumina). Approximately 300 million reads were obtained for each sample. The software Barcode Deconvoluter (Cellecta) was used to convert sequencing results to the number of reads for individual barcode sequences, which corresponds to the number of cells expressing each shRNA construct. Fold change (FC) was obtained for each shRNA by dividing reads per million (RPM) in the pomalidomide-treated sample by RPM in the control sample. FC is the measure of change in pomalidomide sensitivity due to each individual shRNA. If a given shRNA is unrelated to the efficacy of the drug, the FC value will be close to 1; if a given shRNA confers resistance to pomalidomide, the FC value will be greater than 1; and if a given shRNA confers hypersensitivity to pomalidomide, the FC value will be less than 1. From the FCs, maxFC and FDR were calculated for each gene. maxFC is the FC with the largest deviation from 1 among FC values of ~5 shRNAs targeting a given gene. On the other hand, FDR is based on all FC values associated with a given gene, and was obtained using a modified rank product method (see below). Genes with |log_2_(maxFC)| > log_2_(3) or FDR < 0.1 were identified as pomalidomide-affecting genes.

The rank product method, a nonparametric method originally developed for the detection of differentially expressed genes in replicated microarray data^31^, was modified to allow analysis of the screening results. First, shRNAs were ranked in descending or ascending order of FCs in each replicate, and *Rij* was defined as the rank of the *i*^th^ shRNA in the *j*^th^ replicated experiment. The rank product statistic for the *i*^th^ shRNA was defined as the geometric mean of *Rij* obtained from *K* replicated experiments, as follows:

$$R{P}_{i}={(\mathop{\prod }\limits_{j=1}^{K}{R}_{ij})}^{1/K}$$

The rank product statistic for a gene (*RPG*) was then defined as the geometric mean of all the rank products of shRNAs targeting the same gene, as follows:

$$RPG={(\mathop{\prod }\limits_{i=1}^{x}R{P}_{i})}^{1/x}$$

Random permutation testing with 100 iterations was applied to assess the significance of each *RPG*, and the average expected value and the FDR were estimated for each gene according to Breitling *et al*.^31^.

To identify biological processes associated with the 445 genes identified by the screen, GO enrichment analysis was carried out using DAVID v. 6.8^32,33^. GO Biological Process terms with p-value < 0.01 were considered significant and are shown in Table 1.

### Gene knockdown using individual shRNAs

A pRSI9-based control vector was prepared by removing the barcode and shRNA sequence from a pRSI9 plasmid isolated from the libraries. The resultant pRSI9 control vector carries the puromycin resistance gene and RFP. Synthetic double-stranded oligonucleotides encoding shRNA, listed in Supplementary Table 3, were cloned into the control vector. Recombinant lentiviruses were prepared using the resultant plasmids and the ViraPower Lentiviral Packaging Mix (Thermo Fisher Scientific), and were transduced into OPM-2 cells to achieve stable knockdown. For transient knockdown, 293T cells were transfected with a control or shRNA-containing plasmid using Lipofectamine 2000 (Thermo Fisher Scientific). pRSI9_eGFP was generated by replacing RFP with GFP and used for the competition assay (see below).

### Immunoblotting

Cell lysates were prepared with high salt lysis buffer (150 mM Tris-HCl [pH 7.9], 500 mM NaCl, 1% (w/v) Nonidet P-40). Immunoblotting was performed according to a standard procedure using the following primary antibodies: anti-GST (sc-138, Santa Cruz Biotechnology), anti-β-actin (ab6276, Abcam), anti-Aiolos (sc-101982, Santa Cruz Biotechnology), anti-GSPT1 (ab49878, Abcam), anti-DDB1 (ab97522, Abcam), anti-CUL4A (ab72548, Abcam), anti-ROC1 (ab86862, Abcam), and anti-CRBN (CRBN65 mAb, Celgene)^34^.

### RNA preparation and quantitative RT-PCR

Total RNA was prepared using Sepasol-RNA I Super G (Nacalai Tesque). Quantitative RT-PCR analyses were performed using the One Step SYBR PrimeScript RT-PCR kit (Takara) and primers listed in Supplementary Table 4 on a StepOnePlus real-time PCR system (Thermo Fisher Scientific). *GAPDH* was used as an internal control.

### Cell viability assay

SF assay and competition assay were used to investigate the effect of pomalidomide on cell proliferation. For the SF assay, CC-885-treated or untreated cells were plated in 96-well plates and incubated with Cell Count Reagent SF (Nacalai Tesque) for 1 h. Then, relative cell numbers were estimated by measuring the absorbance at 450 nm. For the competition assay, an equal number of shRNA-transduced RFP-positive cells and control GFP-positive cells were mixed and incubated with pomalidomide for 4 or 6 days. Then, the number of respective cells was quantified using a Tali image-based cytometer (Thermo Fisher Scientific), and the resistance index was calculated as described in Fig. 2a.

### Immunofluorescence

Cells were fixed with 4% paraformaldehyde in PBS, permeabilized with 0.4% Nonidet P-40 in PBS, and blocked with Blocking One (Nacalai Tesque). Blocking One was also used for dilution of antibodies. The following primary antibodies were used: anti-HA (901501, BioLegend), anti-Aiolos (sc-101982, Santa Cruz Biotechnology), anti-IRF4 (sc-6059, Santa Cruz Biotechnology), and anti-GSPT1 (ab49878, Abcam).

## Supplementary information

Supplementary Tables.

Supplementary Figures.

## Data Availability

Essentially all data generated or analyzed during this study are included in this article and its supplementary information files. Additional data that support the findings of this study are available from the corresponding author upon reasonable request.

## References

[1] Ito T (2010). Identification of a primary target of thalidomide teratogenicity. Science.

[2] Melchert M, List A (2007). The thalidomide saga. Int. J. Biochem. Cell Biol..

[3] Matyskiela ME (2016). A novel cereblon modulator recruits GSPT1 to the CRL4 CRBN ubiquitin ligase. Nature.

[4] Zhu YX (2011). Cereblon expression is required for the antimyeloma activity of lenalidomide and pomalidomide. Blood.

[5] Lopez-Girona A (2012). Cereblon is a direct protein target for immunomodulatory and antiproliferative activities of lenalidomide and pomalidomide. Leukemia.

[6] Soucy TA, Dick LR, Smith PG, Milhollen MA, Brownell JE (2010). The NEDD8 Conjugation Pathway and Its Relevance in Cancer Biology and Therapy. Genes Cancer.

[7] Cope GA, Deshaies RJ (2003). COP9 signalosome: A multifunctional regulator of SCF and other cullin-based ubiquitin ligases. Cell.

[8] Krönke J (2014). Lenalidomide causes selective degradation of IKZF1 and IKZF3 in multiple myeloma cells. Science.

[9] Lu G (2013). The Myeloma Drug Lenalidomide Promotes the Cereblon-Dependent Destruction of Ikaros Proteins. Science.

[10] Gandhi AK (2013). Immunomodulatory agents lenalidomide and pomalidomide co-stimulate T cells by inducing degradation of T cell repressors Ikaros and Aiolos via modulation of the E3 ubiquitin ligase complex CRL4 CRBN. Br. J. Haematol..

[11] Krönke, J. *et al*. Lenalidomide induces ubiquitination and degradation of CK1α in del(5q) MDS. *Nature* **523** (2015). 10.1038/nature14610 PMC4853910 26131937

[12] Petzold G, Fischer ES, Thomä NH (2016). Structural basis of lenalidomide-induced CK1α degradation by the CRL4CRBN ubiquitin ligase. Nature.

[13] Donovan KA (2018). Thalidomide promotes degradation of SALL4, a transcription factor implicated in Duane Radial Ray syndrome. Elife.

[14] Matyskiela ME (2018). SALL4 mediates teratogenicity as a thalidomide-dependent cereblon substrate. Nat. Chem. Biol..

[15] Asatsuma-Okumura Tomoko, Ando Hideki, De Simone Marco, Yamamoto Junichi, Sato Tomomi, Shimizu Nobuyuki, Asakawa Kazuhide, Yamaguchi Yuki, Ito Takumi, Guerrini Luisa, Handa Hiroshi (2019). p63 is a cereblon substrate involved in thalidomide teratogenicity. Nature Chemical Biology.

[16] Ito T, Handa H (2016). Cereblon and its downstream substrates as molecular targets of immunomodulatory drugs. Int. J. Hematol..

[17] Sievers QL, Gasser JA, Cowley GS, Fischer ES, Ebert BL (2018). Genome-wide screen identifies cullin-RING ligase machinery required for lenalidomide-dependent CRL4CRBN activity. Blood.

[18] Patil A, Manzano M, Gottwein E (2019). Genome-wide CRISPR screens reveal genetic mediators of cereblon modulator toxicity in primary effusion lymphoma. Blood Adv..

[19] Ström, A. C. & Weis, K. Importin-beta-like nuclear transport receptors. *Genome Biol*. **2**, reviews 3008.1–3008.9 (2001). 10.1186/gb-2001-2-6-reviews3008 PMC138946 11423015

[20] Kalderon D, Roberts BL, Richardson WD, Smith AE (1984). A short amino acid sequence able to specify nuclear location. Cell.

[21] Fornerod M, Ohno M, Yoshida M, Mattaj IW (1997). CRM1 is an export receptor for leucine-rich nuclear export signals. Cell.

[22] Nakai K, Horton P (1999). PSORT: a program for detecting the sorting signals of proteins and predicting their subcellular localization. Trends Biochem. Sci..

[23] Alekseev S (2008). Cellular concentrations of DDB2 regulate dynamic binding of DDB1 at UV-induced DNA damage. Mol. Cell. Biol..

[24] Nguyen TV (2016). Glutamine Triggers Acetylation-Dependent Degradation of Glutamine Synthetase via the Thalidomide Receptor Cereblon. Mol. Cell.

[25] Fischer ES (2014). Structure of the DDB1-CRBN E3 ubiquitin ligase in complex with thalidomide. Nature.

[26] Liu J (2014). CRL4ACRBN E3 ubiquitin ligase restricts BK channel activity and prevents epileptogenesis. Nat. Commun..

[27] Chen Y-A (2015). The Cullin 4A/B-DDB1-Cereblon E3 ubiquitin ligase complex mediates the degradation of CLC-1 chloride channels. Sci. Rep..

[28] Kwon E, Li X, Deng Y, Chang HW, Kim DY (2019). AMPK is down-regulated by the CRL4A-CRBN axis through the polyubiquitination of AMPKα isoforms. FASEB J..

[29] Chen ZS, Tiwari AK (2011). Multidrug resistance proteins (MRPs/ABCCs) in cancer chemotherapy and genetic diseases. FEBS J..

[30] Agapova OA (2013). Multiplex Genome Engineering Using CRISPR/Cas Systems. Science.

[31] Breitling R, Armengaud P, Amtmann A, Herzyk P (2004). Rank products: a simple, yet powerful, new method to detect differentially regulated genes in replicated microarray experiments. FEBS Lett..

[32] Huang DW, Sherman BT, Lempicki RA (2009). Systematic and integrative analysis of large gene lists using DAVID Bioinformatics Resources. Nat. Protoc..

[33] Huang DW, Sherman BT, Lempicki RA (2009). Bioinformatics enrichment tools: paths toward the comprehensive functional analysis of large gene lists. Nucleic Acids Res..

[34] Ren Y (2016). A dual color immunohistochemistry assay for measurement of cereblon in multiple myeloma patient samples. Appl. Immunohistochem. Mol. Morphol..

