# Supplementary material for: Genome-wide screening reveals a role for subcellular localization of CRBN in the anti-myeloma activity of pomalidomide
Source: Sci Rep. 2020 Mar 4;10:4012. doi: 10.1038/s41598-020-61027-w (PMC7055313; doi:10.1038/s41598-020-61027-w)
Supplement: Supplementary file 2 — Supplementary Figures. [file 41598_2020_61027_MOESM2_ESM.pdf]

Genome-wide screening reveals a role for subcellular localization of CRBN in the anti-myeloma activity of pomalidomide

Shumpei Tateno[1], Midori Iida[2], Satoshi Fujii[2], Tetsufumi Suwa[1], Miki Katayama[1], Haruka Tokuyama[1], Junichi Yamamoto[3], Takumi Ito[3], Satoshi Sakamoto[1], Hiroshi Handa[3] & Yuki Yamaguchi[1*]

[1]School of Life Science and Technology, Tokyo Institute of Technology, Yokohama 226-8501, Japan
[2]School of Computer Science and Systems Engineering, Kyushu Institute of Technology, Iizuka 820-0067, Japan
[3]Department of Chemical Biology, Tokyo Medical University, Shinjuku 160-8402, Japan

[*]Corresponding author
Phone: +81-45-924-5798, Fax: +81-45-924-5834, E-mail: yyamaguc@bio.titech.ac.jp

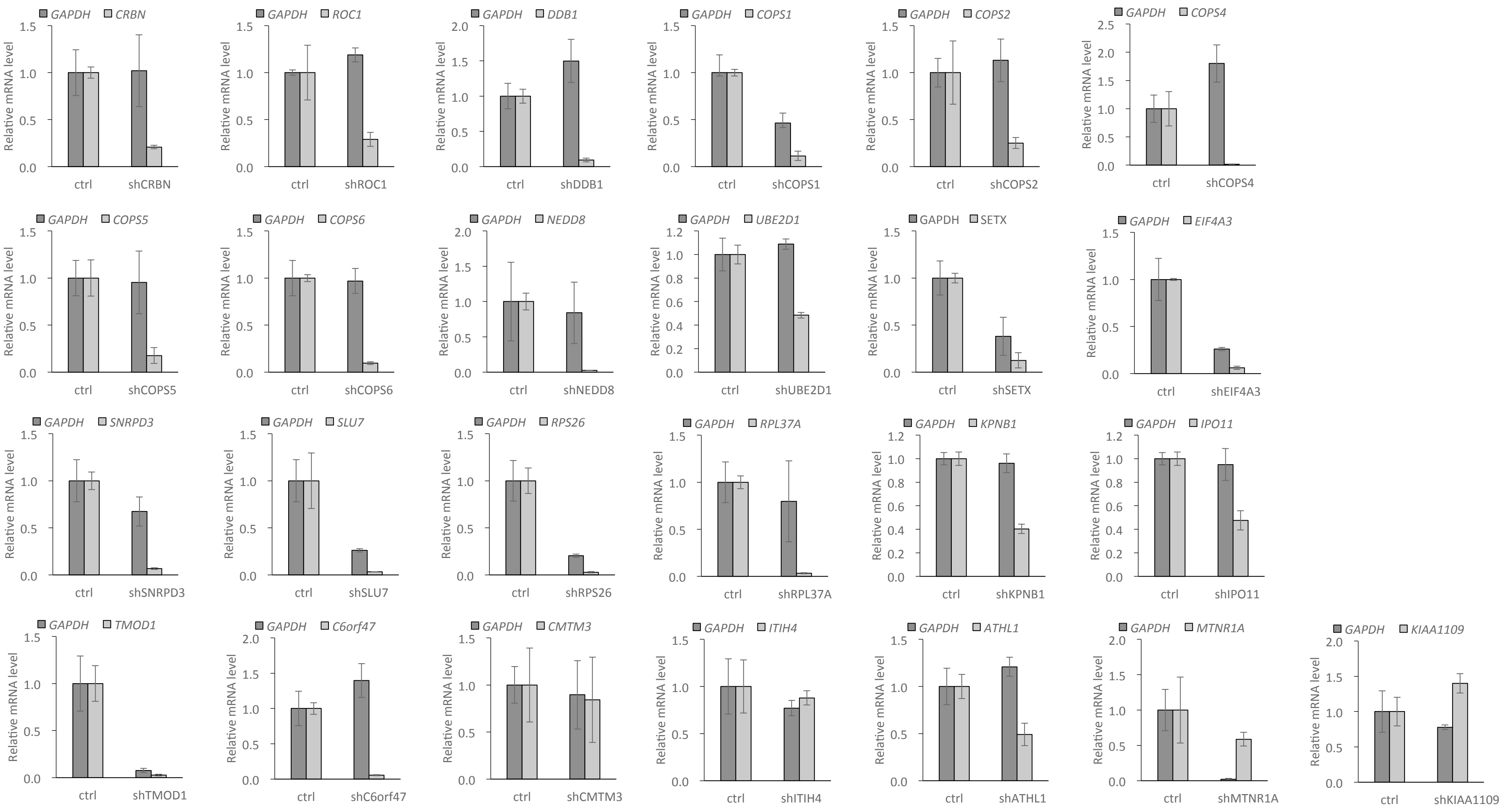

Supplementary Figure 1. Quantitative RT-PCR results showing knockdown efficiency of the genes analyzed in Fig. 2. Several days after lentiviral transduction of respective shRNAs into OPM-2 cells, the mRNA levels of *GAPDH* and shRNA target genes were quantified. Data represent means ± S.D. (n = 3). In some cases, expression of *GAPDH* was substantially reduced; this is most likely due to growth defects caused by knockdown of shRNA target genes. Expression of *SNU13*, *PHF5A*, *ABCC12*, and *OR7G3* was not successfully detected by the analysis.

Fig. 3d

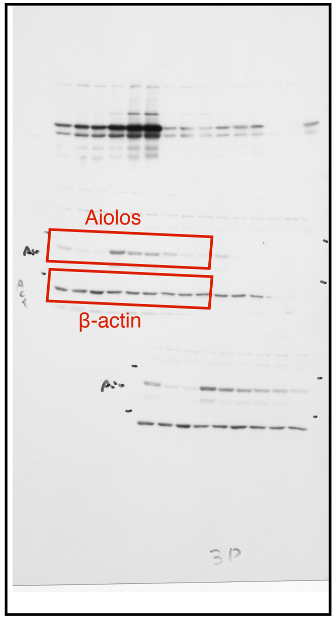

Fig. 3e

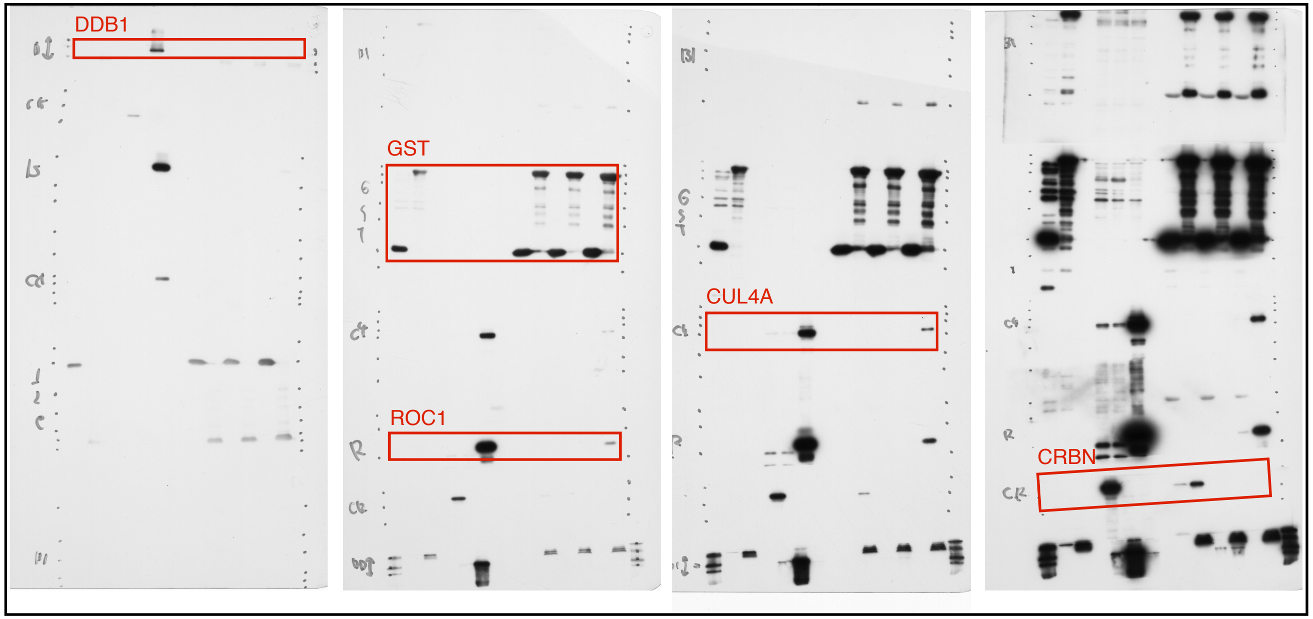

Fig. 4a

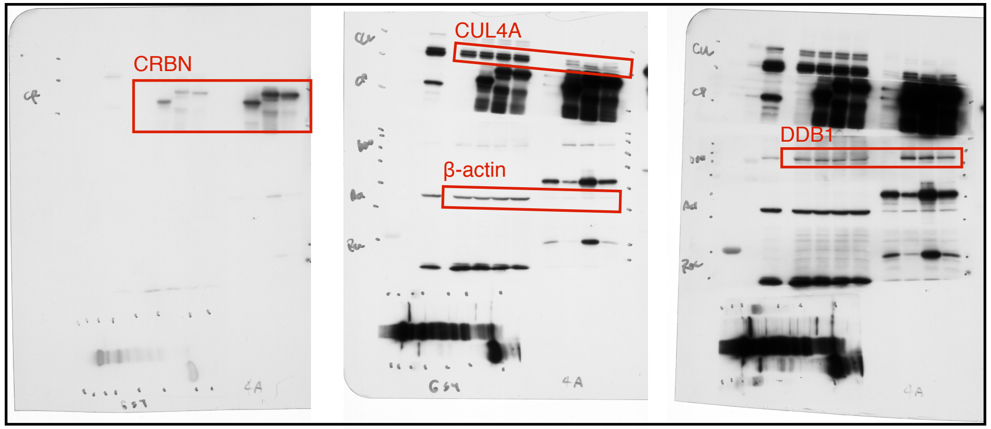

Fig. 4c

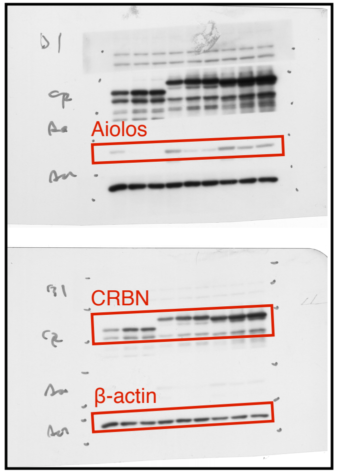

Fig. 4d

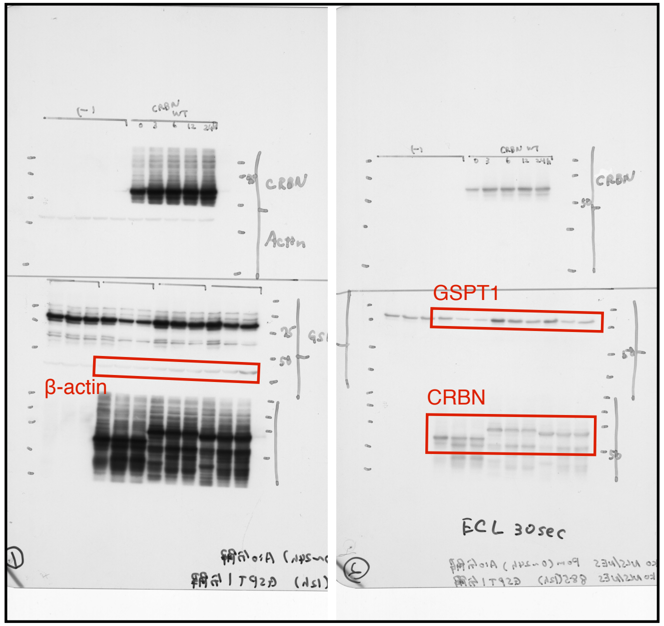

Supplementary Figure 2. Original scanned images of the immunoblots shown in Fig. 3 and 4.
